# A General Framework for Making Context-Recognition Systems More Energy Efficient †

**DOI:** 10.3390/s21030766

**Published:** 2021-01-24

**Authors:** Vito Janko, Mitja Luštrek

**Affiliations:** Department of Intelligent Systems, Jozef Stefan Institute, 1000 Ljubljana, Slovenia; mitja.lustrek@ijs.si

**Keywords:** context recognition, optimization, modeling, energy efficiency, Markov chains, duty cycling, decision-trees

## Abstract

Context recognition using wearable devices is a mature research area, but one of the biggest issues it faces is the high energy consumption of the device that is sensing and processing the data. In this work we propose three different methods for optimizing its energy use. We also show how to combine all three methods to further increase the energy savings. The methods work by adapting system settings (sensors used, sampling frequency, duty cycling, etc.) to both the detected context and directly to the sensor data. This is done by mathematically modeling the influence of different system settings and using multiobjective optimization to find the best ones. The proposed methodology is tested on four different context-recognition tasks where we show that it can generate accurate energy-efficient solutions—in one case reducing energy consumption by 95% in exchange for only four percentage points of accuracy. We also show that the method is general, requires next to no expert knowledge about the domain being optimized, and that it outperforms two approaches from the related work.

## 1. Introduction

In recent years, we have experienced a rise in the popularity of smart wearable devices. Practically everyone owns a smartphone, with other devices like smart watches slowly following this trend [1]. All these devices can use sensors to track the users who wear them, detecting everything from their heart rate and movement to their location. Collected sensor data can then be processed and interpreted using artificial-intelligence methods. We will call this task of transforming sensor time-series data into something semantically relevant about the user *context recognition*. For example, by using a single sensor like an accelerometer, we can detect many locomotion activities, e.g., walking, running, or counting the number of steps the user has made. If we have multiple different sensors we can detect even more complex contexts, such as traveling, working, or eating at a restaurant.

Context-recognition applications can be very convenient for the user, e.g., motivating them to exercise, helping them navigate, changing their smartphone settings based on their surroundings, etc. In some cases, they may also be used to assist their health management by monitoring their physiological signals, analyzing their activity patterns, or even detecting if they fell and injured themselves. As a result, they are widely adopted [2,3,4] and often come preinstalled on wearable devices.

A major limitation of continuous sensing and context recognition, however, is the increased battery consumption of the device that is collecting and possibly processing the data. It is easy to see that if an application halves the battery life of a smartphone, it will probably not be adopted by many users, regardless of its context-recognition ability. The same problem occurs with basically any other wearable device, making the energy-efficiency one of the most important—yet often neglected—aspects of the design of a context recognition system.

Some solutions for conserving the battery life of a device try to reduce the energy consumption by finding the ideal sampling rate of sensors or by finding a good schedule for turning them on and off periodically (duty cycling). Finding the best such sensor setting for a particular context-recognition application is already a challenging task, but a single setting for the whole application might be suboptimal. For example, it might be useful to have the GPS active while the user is driving, but accelerometer could be more suitable when they are indoors. This illustrates the need for dynamically changing sensor settings based on the user’s current situation.

Many adaptive approaches already exist and a comprehensive review of them can be found in Section 2. Most of these approaches, however, rely on having expert knowledge of the domain. For example, in the work of Yi et al. [5], they propose to create a flowchart with hand-picked states, stating for each state what sensor to use and under which condition to transition into another state. An obvious problem with such approaches is that they are domain specific, and when trying to recreate them for a different domain one would need a lot of domain knowledge and/or experimentation.

Other proposed approaches are specialized and can only optimize a particular type of settings (e.g., sampling frequency). Such approaches then obviously cannot be reused to optimize a different type of settings (e.g., which sensors to use). It would be useful to have a general method that can be provided with a context-recognition dataset and an indication of what parameters can be optimized and from this generate dynamic energy-efficient solutions.

A relatively simple and general solution for the above problem would be to select the best sensor setting for each context, and then use it when such a context is detected. However, since such selection is made for each context in isolation, the effect on the whole system could be unpredictable. To illustrate: we could determine that the accelerometer is the best sensor to detect walking, while the GPS is the best at detecting driving. However, by using only an accelerometer when walking we might never detect driving, and the sensor switch might never occur.

The problem becomes even harder if we want to adapt system settings not only to the current context but also to the underlying data used for classification. If, for example, the current user of the system is connected to a Wi-Fi access point, their location could be inferred from the access point ID. If not, other sensors must be used for the task. This opens up an enormous search space of possible energy-efficient solutions, as the data (or the features calculated from it) can have many possible values. Traversing this search space and finding sensible feature values to adapt to—especially without using expert knowledge—is thus a very challenging task.

In this paper we present three different methods for generating energy-efficient solutions. The first two were already presented in our previous work [6,7] and are here briefly described, while the third one is entirely novel. In addition, we show how to combine all three of them to get further energy savings. These methods optimize the energy efficiency in different ways and are designed to complement each other.

In summary, our methodology uses mathematical models to provide an accurate estimate of the system behavior under different configurations—taking into account possible context switches and misclassifications—and then uses multiobjective optimization to find good energy-efficient solutions. It aims to generate the whole Pareto-optimal set of different trade-offs between the energy consumption and classification quality, giving the system designer the possibility to make an educated choice between them and choose one that most suits the application needs.

They all attempt to create good solutions for an arbitrary context-recognition problem, without any expert knowledge of the domain, and two of them have no limitations on what settings are being optimized. Furthermore, they require almost no hand-picked parameters and work with an almost arbitrary function for evaluating either the system quality or its energy consumption. Finally, the implementation of this methodology is openly available in the form of a Python library [8].

## 2. Related Work

In this section we will first describe some of the different system settings that can be optimized and some heuristics on how to optimize them (Section 2.1). Then in Section 2.2 we present some existing methodologies on how to make the system adapt—dynamically change these settings—based on a variety of factors.

### 2.1. Optimization Modalities

The first common setting to be optimized is the sensor sampling frequency. Ideally, the sampling frequency should be high enough to capture the relevant aspect of the data but not any higher. It was shown in several domains [9,10] that the energy required increases almost linearly with the increasing frequency, but the classification accuracy usually increases rapidly up to a certain point, and then reaches a plateau and stops improving. It has been empirically established that the frequency of 20 Hz is required and sufficient for many context-recognition tasks [11,12] and can be used as a first approximation when choosing a suitable frequency. This is, however, not always the case [13,14]. This “ideal” sampling frequency can be determined in several different ways. One solution is collecting high-frequency data and then iteratively downsampling it, testing the classification accuracy at each step [10,15]. A different, statistical approach (using Kolmogorov–Smirnov statistical test) that is not dependent on either the context-recognition task or the classification model is presented in the work of Khan et al. [13]. It is also of note that different features [16] or classifiers [17] may be more or less dependent on the sampling frequency.

Another very common setting is the duty cycling. The majority of contexts that we may be interested in change only infrequently. For example, user activities such as walking or resting usually persist for a few minutes before changing, while contexts like home or work could persist for hours before changing. This naturally leads to the idea of determining the context and then turning sensors and subsequent processing off for a given time before turning them on again. During the off (also called sleeping) cycle, an application usually assumes that the context has not changed. As a concrete example of the benefits of duty cycling, Bosch et al. [18] were measuring the level of a person’s activity and using them managed to preserve 60% of battery life in exchange for five percentage points accuracy loss. Note that instead of constantly using only a fixed duty-cycle length, many approaches [19,20] opt for changing the duty-cycle lengths based on a variety of factors (e.g., the last classified context) as explained in Section 2.2.

The third common approach for decreasing the energy consumption is to change the sensors used for the recognition task. For example, location can be accurately determined by using the GPS, but it can also be determined (perhaps with a slightly lesser accuracy) using the Wi-Fi signal [21]—consuming four times less energy [22,23]. Alternatively, the location could be determined by location fingerprinting using ambient sound, light, and color [24] or by getting the location information from another device that may be connected to it [25]. The choice of sensors can thus affect both the energy consumption and classification accuracy. In general there is no mathematical (domain-knowledge-free) method to determine which sensors can be replaced with which, without first recording a dataset using those sensors in addition to analyzing their energy requirements.

### 2.2. Dynamically Adapting Sensor Settings

Adapting the system settings to the circumstances (e.g., current context, feature values, context stability, etc.) is the core of this research area, as there are many different factors the settings can adapt to and many methods for deciding how to adapt.

In some domains expert knowledge suffices to design a useful sensor adaptation scheme. An example can be found in the work of Wang et al. [5], where they used an xml scheme of instructions to determine when to use a specific sensor. Such a scheme can be very flexible and able to adapt to different types of factors (properties of the data, feature values, classified context, etc.). Many domain-specific approaches [26] would fall into this category.

A big category of approaches is based on the last recognized context. Each context has a setting assigned and that setting is used whenever the corresponding context is recognized. Deciding which settings to use for which context, however, is nontrivial. Even if both the set of contexts and the set of settings are fixed, the problem exhibits a combinatorial explosion of possibilities. In fact, it can be proven that finding the optimal assignment is at least NP-hard [6]. Approximate methods are thus required.

A simple example of such approximation is to decide for each activity in isolation which setting is best for that activity [15,27]. This is done by testing each activity against each possible setting, and determining, for example, that sampling frequency does not affect the recognition of *resting*, but it needs to be high to detect *running*. In this example, a high sampling frequency would be assigned to *running* and low to *resting*. This greatly reduces the complexity of the search, but it may be a poor approximation for the performance of the system. What often happens in practice, but is not modeled by this method, is that a misclassification causes a switch to a setting inappropriate for the current context, which in turn leads to further misclassifications.

A way to partially mitigate the described problem is to modify the system in a way so that whenever a context change is detected, a “maximum” (all sensors, max frequency, etc.) setting is used to determine the correct context [28]. After that, an appropriate setting for that context is used as usual. This helps to mitigate misclassification chains but in turn has the downside of using the “maximum” setting often, especially in a domain where either frequent misclassifications or frequent context changes are expected.

Another variation is to base the sensor settings on the context that system believes would happen next, instead of the current one. In the work of Gordon et al. [29], the next most probable contexts were predicted using a Markov chain model, and then a sensor subset that can best distinguish between them was used. The best subset is again determined in isolation and thus has all the above-described problems. Nonetheless, they reduced the energy consumption when working with the publicly available Opportunity dataset [30] by 90% in exchange for three percentage points of accuracy.

An altogether different way of using context for adapting the system settings is tracking the context change. If the context is “stable” and has remained unchanged for a while, sensing capabilities could be lowered. This approach is particularly useful in combination with duty cycling. When the context is rapidly changing, a short duty cycle is used; when the context is stable, an increasingly longer duty cycle is used instead [19,20,31,32]. In the work of Au et al. [32] the duty cycle was lengthened linearly while the context was stable, but its length was divided by a constant otherwise. Rachuri et al. proposed a similar scheme, but they were multiplying the duty-cycle length in the case of the stable context. Sarker et al. [31] also proposed multiplying but with a relatively complex formula. In all cases, the minimum and the maximum duty-cycle length was bounded. A combination of this and the previous approach can be found in the work of Lee et al. [10].

Context-based approaches are convenient as the number of possible contexts is usually limited. On the other hand, it restricts the adaptation to the context itself. Imagine having an application that recognizes the words people are speaking around it and wanting to avoid using a complex processing pipeline when the surroundings are totally silent. Silence is an attribute of the data and not a recognized context, hence no approach in the previous section could model this behavior. This example in fact represents the most frequent use of this adaptation method. System sensing capabilities lower when the incoming data is in a way “uninteresting”. Typical indicators are sound sensors hearing silence [33], movement sensors detecting no movement [34], or location sensor detecting no location change [21]. A different type of “uninteresting” is presented in the work of Lu et al. [33], where the data is discarded if it is too similar to the previously acquired (implying that the context has not changed). The last listed approach in fact used several different techniques (different for each sensor) in a complex pipeline. To make the system more robust, the setting is sometimes [34] only changed if the “uninteresting” data persists for a longer time, or alternatively, the systems settings are gradually lowered [33] in that case. Such systems can be simple to implement and usually work well in practice but are inflexible as they can only adapt to some predefined notion of “uninteresting” data.

Another similar category of approaches uses a specific data pattern as a trigger. For example, one may turn the GPS on only when the accelerometer detected movement [35]. Similarly, Park et al. [36] used a gyroscope in their work only when the accelerometer detects data that could be a part of a hand gesture. In the work of Rahmati et al. [37], a combination of acceleration data, time, and available cellular towers was fed to a mathematical model to determine the probability of Wi-Fi being available, without turning on its interface. The RAPS [38] system decides which location sensors to use based on which were needed the last time the user was around this location at a similar point of day. Doing so, they managed to reduce the battery consumption by a factor of four, while retaining their localization accuracy.

Most approaches presented so far rely heavily on expert knowledge. Adapting to the data, using a domain-knowledge-free method is done less frequently. The most popular way of doing so is using a Markov decision process [39,40,41] that models which setting to use given the current state. States are usually represented by discretizing different features, while possible actions are defined as the possible settings to use for the next instance. In the work of Au et al. [40] the states were defined with the help of expert knowledge, while in the work of Cheng et al. [39], all features and the last predicted context were taken as a possible state. This last approach thus implicitly combines the adaptation to data and context. An alternative is to use cost-sensitive learning methodology which is used for similar tasks in other domains and was adapted for context-recognition in our previous work [42].

## 3. Methodology

In this section we present three different methods for energy-efficient context recognition and then show how to combine them to join their strengths. The first two adapt system settings to the current context, while the last one adapts them directly to the sensor data.

For the first two methods—named Setting-to-Context Assignment (SCA) and Duty-Cycle Assignment (DCA)—we assume that we have a system that works as follows: the system is in one of the predefined contexts (e.g., the user’s activity), and the goal of the system is to recognize it. Each context is associated with some setting. For the SCA method (Section 3.1), the settings can be anything—e.g., which sensors are in use, or what sampling frequency is used. For the DCA method (Section 3.2), only duty-cycling settings are considered. Whenever the context-recognition system detects a new context, the setting used switches to the one associated with that context. That setting is then used until a different context is detected. An example for system behavior might go like this: when the user is walking, use low sensor sampling frequency, but when running is detected, use high sensor sampling frequency.

DCA specialization in duty cycling allows for more powerful modeling—an entirely different mathematical model is built—which allows for a more efficient search of this particular optimization modality. Duty cycles were chosen for this special treatment as they can be used in virtually any context-recognition environment and often contribute the most to the energy savings.

The drawback of the previous two discussed methods is that they can only create systems that adapt to the current context (classification class of the system) but not to the actual feature values. The last method—Model Trees (MT)—described in Section 3.3 tries to remedy this by splitting the feature space into different sections using a shallow Model Tree, which in this case is a decision tree that has classification models in its leaf nodes. These classifiers use different system settings, usually different features that require different sensors.

Finally, in Section 3.4 we adapt the context recognition system to both the underlying data (expressed by features) and the context at the same time by combining all three methods. Doing so combines the advantages of each method and usually generates the most energy-efficient solutions.

### 3.1. Setting-to-Context Assignment (SCA)

Suppose we have a sensing system that works as previously described: the user is in one of the predefined contexts c∈C (e.g., the user’s activity), and the goal of the system is to recognize it. To each context we assign some setting s∈S and that setting is used whenever that context is detected.

Given possible settings and contexts, the main task is to efficiently find a setting-to-context assignment a:C↦S that generates good trade-offs between the system energy consumption and its classification quality (for example: the accuracy of the sensing system). What trade-off is the most suitable for a given domain is up to the system’s designer, but instead of forcing them to choose an arbitrary weight between the two criteria, we should try to present them the Pareto-optimal solutions to choose from. A solution is considered Pareto-optimal if there is no other solution that dominates it, i.e., that is better in one of the two objectives and at least as good in the other one.

A guaranteed way to find the Pareto-optimal set of assignments would be to determine the performance of each one by testing them on the collected dataset, and then removing all assignments whose performance is dominated by another assignment. This approach of course quickly becomes infeasible when increasing the number of settings or contexts, as the number of assignments becomes exponentially large: if we have $\left|C\right|$ contexts and $\left|S\right|$ settings, we have ${\left|S\right|}^{\left|C\right|}$ different assignments. As an example from the Commodity12 dataset (Section 4.3): having 10 sensors that can be either on or off gives us $\left|S\right|=2^{10}=1024$ different settings. With 7 different contexts the total number of assignments becomes $1024^{7}\approx 10^{21}$. A second, additional, problem is the time of performance evaluation for even one single assignment. One must traverse the dataset, simulating different settings and classifying instances one by one in order to determine which setting to use for the next one—a process that can be prohibitively slow if we plan to evaluate a large number of assignments. In the Commodity12 example, the evaluation of one assignment lasts a couple of seconds (model training time not included) on standard hardware, which still results in the optimization process that can last several days even if only a small subset of all possible assignments is tried.

A common alternative solution would be to use domain knowledge to determine a small number of sensible assignments and test only those. However, such knowledge is often either not available or not good enough to isolate all good potential assignments, which calls for an automatic method for solving the task. In Section 3.1.1 and Section 3.1.2 we show how to reduce the evaluation time of a single assignment and then in Section 3.1.3 how to select a subset of assignments to evaluate.

#### 3.1.1. Simple Evaluation Model

The goal is to mathematically model an assignment in order to be able to predict its performance without doing actual experiments—or at least minimizing the required number of them. Doing so allows us to test more assignments and gives us better odds at finding good ones. In this scenario an experiment would be to classify all instances in the dataset while simulating different sensor settings and switching between them based on the assignment.

The simple approach considers each context and its potential settings in isolation. That makes it similar to some methods from the related work [15,28] which also implicitly makes a similar isolated choice. To use it, we first determine the accuracy using each of the settings but without switching between different settings based on the context. This requires $\left|S\right|$ experiments, exponentially fewer than ${\left|S\right|}^{\left|C\right|}$. Then, to evaluate the system accuracy when using a specific assignment *a* we simply take the accuracy of detecting each context *c* using the assigned settings a(c) and weight them by the proportion of that context in the dataset.

(1) $$Accuracy\left(a\right)=\sum_{c\in C}p(c)Acc(c|a(c))$$

a(c)—the setting assigned to context *c*.
p(c)—the probability of context *c*, estimated by its proportion in the dataset.
Acc(c|s)—the accuracy for recognizing context *c*, given the setting *s* is active.

Let us take the example of having two contexts $c_{1}$, $c_{2}$ and two corresponding settings $s_{1}$, $s_{2}$. Additionally, we suppose that using $s_{1}$, we can predict $c_{1}$ with the accuracy of 60%, and using $s_{2}$, we predict $c_{2}$ with the accuracy of 100%, and that both contexts appear an equal amount of time. In this case we can make a prediction that this assignment will produce 80% accurate results. We can make a similar prediction about the energy consumption, given the information about the energy requirement of each setting.

While this evaluation is both simple and intuitive it does not make the most accurate predictions. What happens in practice is that due to the misclassifications, system often switches to a setting not appropriate to the current context which can lead to further errors. In addition, this model does not take into account the times when the context changes and the current setting is not appropriate for detecting the new context. Thus, this model is only presented as a benchmark and as a motivation on why a more powerful model is required.

#### 3.1.2. Markov-Chain Evaluation Model

To improve upon the simple model, we propose [6], a Markov-chain model for making the performance trade-off estimation. A Markov chain is a stochastic process that moves through predefined states, with the probability of every transition being dependent only on the start and end state. To use this model we have to make two assumptions about our problem. First, that the probability of a transition between contexts depends only on the current context (*Markov property*), and second, that the probability of classifying an instance to a context depends only on the current context and the current setting. Both assumptions should hold, at least to a large degree, in most cases of use.

**Figure 1:**
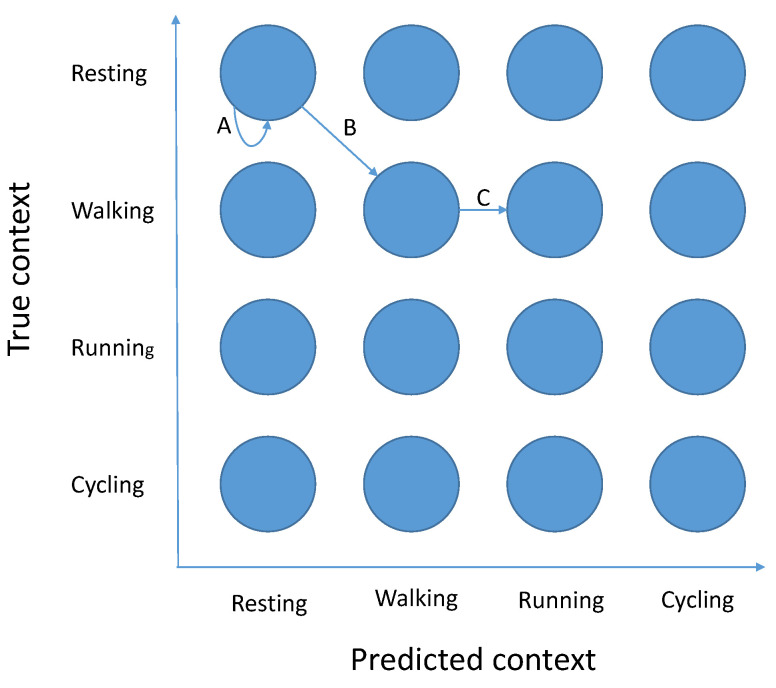
Markov chain where each state represents a 〈true,predicted〉 context. The states are fully connected, including connections to themselves, although most transitions were omitted for clarity. For example, if the user is continually resting and resting is correctly detected, we can represent it with the transition A. Then if the user starts walking, we can note that with transition B. Finally, if the user continues to walk, but the walking is misclassified as running, we represent it with transition C. This example is taken from the E-Gibalec dataset (Section 4.4).

The key insight in creating the Markov-chain evaluation model is to correctly define Markov-chain states, so they (1) contain enough information to calculate transition probabilities between them, and (2) allow us to infer assignment properties from them. To do so we create a Markov chain that has ${\left|C\right|}^{2}$ states. Each state represents a pair (current context, context the system believes we are in), marked $\langle c,c^{\prime }\rangle$ in short. An example of such a Markov chain can be found in Figure 1. The current context (the first part of the pair) is necessary to model how frequent that context is and which contexts are likely to come next. Knowing which context the system believes we are in, or equivalently which context did the system classify the current context to (the second part of the pair), is necessary for modeling which sensor settings are active and thus the system performance using that setting. It is important to emphasize that each assignment would have the same kind of structure but with different transition probabilities between them. The precise procedure on how to calculate the transition probabilities between these Markov states for a particular assignment is described in Appendix A.

Instead of directly traversing the Markov chain (which would be time consuming), we can calculate its *steady state*—that is the proportion of time spent in each of the states given infinite or at least sufficiently long time. Knowing the steady state of the Markov chain is enough to make accurate predictions of the context-recognition system performance, both in terms of its energy consumption and prediction quality.

For example, if one places the state probabilities of the steady state in a table that has the same structure as the states in Figure 1, they would get exactly the confusion matrix of the system that switches settings according to the given assignment. Summing the diagonal would thus yield the accuracy of the system (Equation (2)), but any other metric derived from the confusion matrix (e.g., F-score) could be used instead. Estimating the energy consumption of the system is likewise simple: for each state we know what context was detected and thus what setting is active and how much energy does that setting consume. So by multiplying energy consumption in each state by the proportion of time spent in that state, the total energy consumption is calculated (Equation (3)).

(2) $$Accuracy\left(a\right)=\sum_{c\in M}t(\langle c,c\rangle )$$

(3) $$Energy\left(a\right)=\sum_{\langle c,c^{\prime }\rangle \in M}t\left(\langle c,c^{\prime }\rangle \right)e\left(a\left(c^{\prime }\right)\right)$$

*M*—the set of all states in the Markov chain.
a(c)—the setting assigned to context *c*.
e(s)—the energy requirement of the setting *s* per time unit.
$t(\langle c,c^{\prime }\rangle )$—the predicted proportion of time spent in state $\langle c,c^{\prime }\rangle$, or equivalently, the probability of landing in that state.

#### 3.1.3. Finding Sensible Assignments

In Section 3.1.2 we showed how to estimate the performance of any individual assignment, and Section 5.4 demonstrates that the estimation is both quick and accurate. However, the number of possible assignments is still exponentially large and estimating them all is infeasible. Thus, a more efficient search is required and given that we are essentially solving a problem of multiobjective optimization (the two objectives being the energy efficiency and the classification quality), we can use the methodology from that research field.

We chose the NSGA-II [43], a genetic multiobjective optimization algorithm, for the task. This algorithm is the most widely used for optimizing similar problems with few objectives and we gained good results using it (Section 5.2). Nonetheless, we believe that a different, similar algorithm could be used instead.

The context-to-setting assignments are used as the input to be optimized. They can be evaluated on the fly into energy/quality estimates as needed. This would be excessively time-consuming if we used actual simulation instead of evaluations with the Markov-chain model as hundreds of evaluations have to be performed for each generation in the genetic algorithm.

The encoding of the inputs depends on the settings type and structure. For example, for the Commodity12 dataset (Section 4.3) each setting represented which sensors are turned on or off. This can be encoded by a binary string of length *s*, where *s* is the total number of sensors, e.g., “110000” would represent that only the first two sensors are working. Each context can have a different setting assigned, thus the total length of the encoding is cs, where *c* is the number of contexts, e.g., “110000-100000-00001-00001-00001-00001-00001” represents having only the first two sensors active when the first context is recognized, only the first sensor when the second context is recognized and only the last one otherwise. We used bit-flip mutations and the classical crossover. The same type of representation (with a different number of sensors) was used for the SHL (Section 4.1) dataset. For the E-Gibalec (Section 4.4) and Opportunity (Section 4.2) datasets we enumerated each setting and then used integer vector encoding; e.g., (2, 4, 35, 1) would signify to use the setting that was enumerated as “2” for the first context, setting “4” for the second one and so on. Mutations in this case are the transformation of one vector component into a random value between 0 and the number of settings. Again, the classical crossover was used.

In all cases we used the population of size 100 and let the algorithm run for 500 generations. The crossover probability was set to 0.9 and the mutation probability was set to 0.01 for changing each of the encoded variables. The final solution set includes all nondominated solutions found throughout the entire search, not just those in the final generation. The actual number of nondominated solutions found for each method is listed in Section 5.3. Experimental tests showed that these parameters were adequate for all of our problem domains. As always when using NSGA-II or any similar algorithm, the parameters have to be adapted to each particular problem—and one is encouraged to do so when implementing this methodology on their own domain.

### 3.2. Duty-Cycle-to-Context Assignment (DCA)

Consider a context-recognition system that uses duty cycling with parameters *w* and len. It works for *w* periods, classifying each into one of the contexts c∈C. Then, based on the last classified context *c* it “sleeps” for len(c) periods—turning off all the sensors and classifying all periods as *c* (it assumes that the context does not change while sleeping).

Our first goal is to create a mathematical model that can quickly and accurately predict the energy consumption of such a system and the type of errors it makes due to duty cycling (Section 3.2.1). The second goal is to use this model to find good parameters *w* and len(c) for each *c* (Section 3.2.2).

The main intuition for setting appropriate duty-cycle settings goes as follows: in domains with slowly changing contexts, long duty cycles can be used, while in domains with quickly changing contexts, short duty cycles are required. Note that, if only one of the contexts of interest is short, one might still be inclined to use short duty cycles in order not to miss that context entirely. A good example of the latter is fall detection, where falling is such a short “context” that long sleep cycles should be avoided. The effect of duty cycling can be experimentally determined by discarding the appropriate parts of the data of a recorded dataset. They can also be modeled directly as described in Section 3.2.1.

The problem being solved by the DCA method can be considered similar to the one being solved by the SCA method (Section 3.1), with the exception that all settings are simply the lengths of the duty-cyle phases (*w* and len). Using the SCA, we would start by making an experiment for every (*w*, len) combination in a given range, which can be time consuming given sufficient range. This method leverages the fact that the performance of a setting can be deduced without an experiment. Instead of doing $\left|S\right|$ experiments (one experiment for each setting), only one—the case with no duty cycling—is required. This allows us to evaluate many different duty-cycle parameters and thus increases the chances of finding a good solution.

#### 3.2.1. Modeling Duty-Cycles

To mathematically model duty-cycling we again assume that the dataset has the Markov property, i.e., the next context is dependant only on the current one. In addition, we assume that each misclassification is independent of the others and happens with the likelihood given by a confusion matrix.

Given the assumptions, the evaluation of a system with given duty-cycle parameters can be performed as described in our previous work on the topic [7] and summarized in Appendix B. The main idea is to split duty cycles into cycles of different types, the type being the pair 〈actual context, predicted context〉 just before the sleeping phase. Knowing the predicted context lets us model the length of the sleeping phase, while the actual context lets us model the probability of context transition during the sleeping phase. We can then treat these cycle types as Markov states and estimate the probability of one cycle transitioning into another. Similarly as with the SCA method, we then calculate the steady state of the resulting Markov chain, and from it, the performance of the whole system.

The proposed model consists mainly of multiplications and summations of matrices ($\left|C\right|$ in size) and can evaluate the system given duty-cycle parameters almost instantly—at least as long as the $\left|C\right|$ is reasonably low. In Section 5.4 we show that this is much faster than simulating duty-cycling on the actual dataset, classifying instance by instance.

#### 3.2.2. Finding Duty-Cycle Parameters

The number of possible parameter combinations is typically quite large: $m_{w}m_{s}^{\left|C\right|}$, assuming that the length of the sleeping period is capped at $m_{s}$ and the length of the active period is capped at $m_{w}$. Consequently, despite the speed of the evaluation model, we cannot test every combination with a brute-force approach.

Similarly as when describing the SCA method, we have an optimization problem with two conflicting objectives: reducing the system’s energy requirements, while retaining its classification quality—accuracy/F-score/recall of the most important context or any other metric that can be computed from the confusion matrix of the system. Given the similarity of the problem, we used essentially the same methodology as described in Section 3.1.3: We used NSGA-II [43] algorithm in combination with the previously described mathematical model that can evaluate the performance of an assignment on the fly. The result is once again a nondominated set of solutions, each giving a different trade-off between energy consumption and classification quality, from which the system designer, who knows the requirements of the system, can pick a suitable solution.

The population was encoded as an integer vector, where the *i*-th component signified the length of the sleeping phase of the *i*-th context. The length of the active phase *w* was not included, as the experimental tests confirmed that the length w=1 is ideal [7] (this is not true in the case if there are some real-life restrictions on the sensors—this case is discussed in our previous work [44]). The length of the sleeping phase had an artificial upper bound that was determined independently for each dataset depending on the average lengths of the contexts in it.

### 3.3. Model Trees (MT)

One can imagine that detected contexts divide the feature space into different regions, each requiring different system settings for optimal performance. However, it could easily be the case that each individual context could be further divided into finer-grained regions, especially if the number of contexts is low.

As an example, if the context is *running* and the user is running fast, the motion is easily recognizable using accelerometer alone. If the user is running slowly, however, another sensor (e.g., gyroscope) could be necessary to differentiate it from fast walking. Another example comes from the fall detection problem, where we have only two different contexts: *falling* and *not falling*. In addition to the low number of contexts, the *falling* context is too short to meaningfully change system settings in. It would be more helpful for the system to learn to use more expensive (and accurate) system settings in situations that potentially occur before the fall, e.g., the user is moving fast.

A notable example of a method that iteratively splits the feature space into regions is the classical decision tree. In particular, the Cost-Sensitive Decision Tree (CS-DT) [45] is often used in other domains for this purpose (e.g., deciding which medical tests a patient should undergo, given the results of previous tests). In our previous work [42] we thus tried to adapt the CS-DT methodology for context recognition. Relying on CS-DTs for classification proved to have two problems. First, the constraint of using a decision tree as the classifier, instead of using a potentially more powerful one (e.g., Random Forest or Deep Learning) and second, a CS-DT needs several consecutive instances to detect a context for the first time. To elaborate, the implementation starts by using a sensor as dictated by the root node of the tree, then after the sensor is activated and feature value is determined, the tree is traversed into next node, which dictates which sensor to activate next, all until a leaf node is reached—which is where an accurate classification can be made. This becomes especially problematic if duty cycling is used and sensors are frequently turned off, requiring constant repetition of this process.

To avoid both these issues we here propose a simpler approach that yields better results in practice.

#### Creating Model Trees

The main idea behind the Model Trees (MT) method is to create shallow decision trees—using one or two sensors (we used the tree depth of two layers in our experiments). Each leaf node then gets assigned a classifier (of an arbitrary type) that uses some subset of sensors. The term “Model Trees” [46] usually refers to trees that use linear regressors in its leaves, but our application is similar enough in spirit to justify borrowing the term.

When an instance is being classified, it first descends this shallow MT to determine which classifier the system should use. If all necessary sensors are available, then this classifier is indeed used. If not, this instance is classified using the same classifier as the previous instance and then the appropriate sensors are turned on. It is assumed that sensors used to traverse the MT are always active, hence the recommendation to use as few as possible (with low sampling frequency if possible). This requirement of one sensor always being active is dropped when combining MTs with the SCA method (Section 3.4.2). Thus the sensors used (or a similar setting) adapt to the values of the features chosen by the MT.

To build a MT, the first part is to construct a decision tree: for this part any standard decision tree suffices, given that it allows (or can be modified) for control of the tree depth and that the features used come from a limited number of sensors. The second part is the assignment of classifiers to each of the leaves. Note that the shallowness of the tree ensures that the number of leaves is limited to avoid overfitting. To do so we trained one classifier for each of the possible system settings (similarly to what one would do in the SCA method). We then tested each classifier on instances that would fall in each of the nodes to determine for each node, which classifier fits “best”. Best is here defined as *accuracy* (or some other performance metric) minus *energy cost* weighted by a parameter λ.

In order to avoid hand-picking the λ parameter, we propose to test different values of λ for generating different MTs, then test these MTs and report back the Pareto-front approximation of the achieved trade-offs. As an additional note, each MT is evaluated independently of others MTs, as the MT used is not based on the current context (as opposed to the settings in the SCA/DCA methods). Thus multiobjective optimization is not required and the number of solutions to test is limited. Consequently, empirical evaluation of the dataset suffices and a mathematical model is not needed.

**Figure 2:**
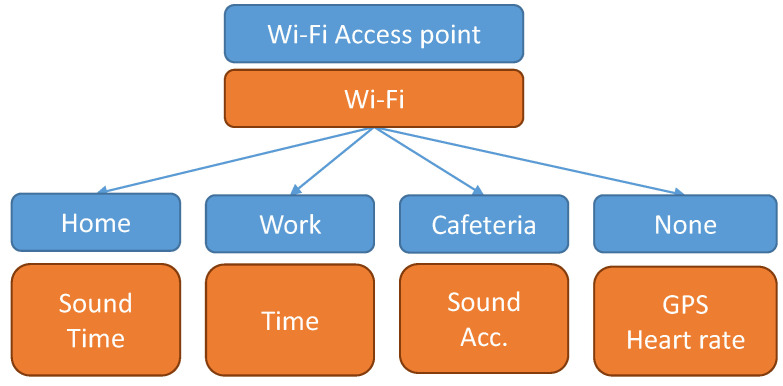
A model tree taken from the Commodity12 dataset. The blue node at the top shows the feature used to create the tree, while other blue nodes represent potential values of this feature. Orange nodes dictate which sensors should be turned on if that node is reached. The sensor in the top orange node is always on.

A potential example of a MT (taken from the Commodity12 dataset, with depth set to one) is shown in Figure 2. Such a MT dictates that Wi-Fi should always be in use while the other sensors should be in use depending on the current access point the Wi-Fi is connected to. For example, if the user is not connected to any Wi-Fi spot, GPS and heart rate sensors are turned on to determine the context. Note that the split made by the tree only partially overlaps with the contexts in this dataset and reveals some additional “regions”—in this case the cafeteria.

### 3.4. Combining Methods

So far, we described three different methods for optimizing the energy consumption, each having their own advantages and disadvantages. The Setting-to-Context Assignment (SCA) method is very general and can work with basically any setting but can only optimize at the granularity level of a context. It requires one experiment for each setting to predict the performance of a system that dynamically switches between them. The Duty-Cycle Assignment (DCA) method negates the last point by mathematically modeling the performance of a setting but does so only for duty-cycle settings. Lastly, the Model Trees (MT) method can optimize at the granularity level of the feature data but not on the context level. To combine the advantages of the methods we propose combining them into one method.

#### 3.4.1. Combining the SCA and DCA Methods

The DCA method assigns a duty-cycle setting to each context. To do so it requires only the transition matrix between contexts and the confusion matrix of the system. As such it can be applied alongside basically any other setting optimization. In this case, one could use the SCA method to optimize all settings but the duty cycling. From the set of trade-offs, a few could be chosen and the confusion matrix of the corresponding system (as calculated by the SCA model) would then used as the input to the DCA method. This way the duty cycling is added to whatever setting optimization was performed by the SCA method with no additional experiments needed. Note that in most practical cases, the optimization of duty cycling is completely orthogonal to the optimization of some other system’s setting.

To make the evaluation of the system performance when using this method combination slightly more accurate, one has to consider an interaction between the two methods. For example, if the SCA method decides to use all sensors for context *a* and only a few sensors for context *b*. Then when context *a* is detected, the duty cycling is more beneficial than when context *b* is detected. The latter follows from the fact that classifications are more costly for the context *a*. To account for this, we can calculate energy consumption in the following way: we multiply the energy consumption of different settings (that are assigned to each context) with the distribution of the corresponding duty-cycle types (Equation (4)). This gets us the average energy consumed when the sensors are active, from which it is easy to calculate the total energy (Equation (5)).

(4) $$ea=\sum_{i=1}^{\left|C\right|}\sum_{j=1}^{\left|C\right|}e\left(a\left(c_{j}\right)\right)D_{i,j}$$

(5) $$Energy=w*ea+len_{avg}*es$$

*D*—the distribution of cycle types. $D_{i,j}$ is the proportion of 〈i,j〉 cycles.

e(s)—the energy consumption of the system setting *s*.

$a(c_{j})$—the setting used when context $c_{j}$ is detected.

*w*—length of the active phase.

$len_{avg}$—the average length of the sleeping phase.

ea—the energy consumption in the active part of the duty cycle.

es—the energy consumption in the sleeping part of the duty cycle. This can be either 0 or the baseline cost of the context-recognition application.

This approach can be even more streamlined by using multiobjective optimization to simultaneously optimize both the SCA and DCA component of the setting. This is achieved by concatenating the settings vectors used for the SCA method with the setting vector used by the DCA method. The fitness function of this concatenated vector is the classification quality/energy achieved when running the two mathematical models, one after another, with the modifications stated in the previous equation.

#### 3.4.2. Combining MT with the Other Methods

The key insight is to imagine different Model Trees (MT) as system settings. If for example, a “regular” setting would be “use accelerometer” or use “gps”, now a setting would be a MT: “use accelerometer if $acc_{x}$ feature value ≥ 0 else use accelerometer and gps”.

Since we can estimate the performance (both in energy and accuracy) of a single MT, we have all prerequisites for using it as a setting for the SCA method. A solution from the SCA method would then be: if *context 1* is detected, use *MT A*, if *context 2* is detected, use *MT B*, etc.

To use MTs as possible system settings, we obviously need to generate many different trees. This can be achieved in two different ways. One is to repeatedly, randomly select the list of features for each tree to select from—the approach used in Random Forest classifier and similar ensemble methods. The other approach is to modify the parameter λ that is determining the trade-off between energy consumption and classification quality. In our experiments we used a combination of both approaches—for each selected feature subset we also tried many different values of λ. The MTs generated in this way were thus different both in which features they use in nodes as well as what energy/quality trade-offs the classifiers in the leaves make.

Using MT as settings thus makes combining the MT and SCA methodology pretty straightforward. The only remaining implementation detail is to decide what setting to use immediately after context change—as we switch to a new MT and need at least one time period in order to determine the branch and thus the setting. We opted to use a classifier that needs only sensors already required for the root MT node.

When adding the DCA method to the MT + SCA combination we need one final adjustment. Since, as discussed before, MTs need more than one time period to decide on the system setting, we also need to have the active period for duty cycling ≥ 2. This limitation can greatly limit the energy-efficiency of duty cycling and can negate the benefits MTs provide.

To counteract this limitation we propose the following implementation: after the sleeping phase is finished, use the “current sensors”, then set the “current sensors” to the senors dictated by the MT. This way an active period of 1 can be used, although the sensors change with a slight delay. We provide no mathematical formula on the effect of such delay but have tested it empirically on the solutions provided by the method combination.

**Figure 3:**
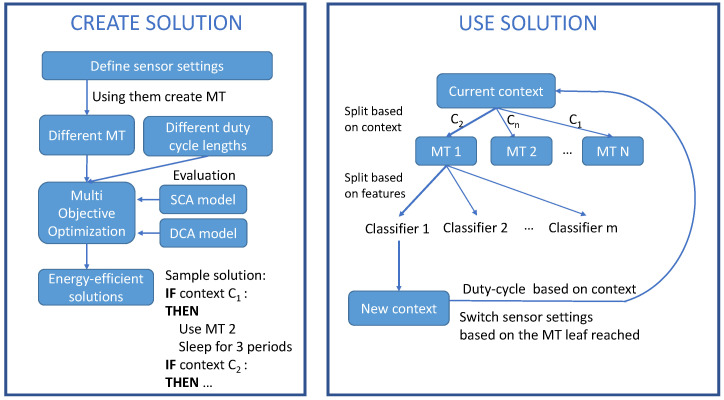
An overview of the three-method combination—both for creating energy-efficient solutions (**left**) and then for implementing one of them on a device (**right**).

A summary of the three-method combination is found in Figure 3.

## 4. Datasets

The presented methodology was tested on four different real-life datasets. They cover a variety of context-recognition tasks from the recognition of basic human activities (walking, running, resting, etc.), high-level activities (work, home, etc.), to very specialized context-recognition tasks (eating, using the fork, etc.) Datasets were collected by attaching various sensors to volunteers and/or their environments. Two datasets used—the Opportunity and SHL—are publicly available and often used for comparing different methods for context recognition.

This section covers each of the datasets in enough detail for the reader to understand their properties and be able to interpret the solutions returned by the presented methods and references a dedicated paper with all the details on how the dataset was collected and processed. In each case we also specify which sensing settings could be modified in order to improve the energy-efficiency of the context-recognition method used for that dataset and how we estimated the energy requirements of these modifications. The summary of the datasets is given in Table 1.

### 4.1. Sussex-Huawei Locomotion (SHL) Dataset

The SHL dataset is one of the largest, publicly available datasets, focused on human activity and locomotion recognition using wearable sensor data. A part of this dataset was used for the 2018 Sussex-Huawei Locomotion Challenge and this subset was also used for our work. The dataset is available on the challenge website [47] and is described in detail in the baseline paper [48].

The participants wore several smartphones on different parts of their body, but in this study we focused on the one worn in their trouser pocket. Data came from the accelerometer, gyroscope, magnetometer, barometer and the orientation of the phone. All sensor data was sampled at 100 Hz.

Altogether, there were 82 recorded days (62 for training, 10 for testing, and 10 for validation). During the recording three participants were tasked with moving around the city and annotating their mode of transportation. The annotated context were *bike*, *bus*, *car*, *run*, *still*, *subway*, *train*, *walk*. Due to the nature of this scenario, the resulting contexts were long on average and had sensible transitions from one to another. In addition, they had a mostly uniform distribution—with the exception of running, which appeared less often.

The full pipeline of the devised context-recognition system can be found in our previous work [49] (this system achieved first place at the SHL Challenge). In summary, we first downsampled the data to 50 Hz to make the system more energy efficient, while retaining the classification accuracy. Next, the data was split into 60-second nonoverlapping windows. Features were calculated separately for each sensor stream (and separately for each axis of a sensor in case of 3 and 4-axial sensors), adding up to a total of 1696 features. The classification was then performed using an ensemble of classifiers: Decision Tree, Random Forest, Naïve Bayes, K-Nearest Neighbours, SVM, Bagging using Decision Trees, Adaptive Boosting, Extreme Gradient Boosting, Multilayer Perceptron, and a Deep Learning model.

As the system setting to be optimized, we took all possible sensor subsets in addition to different duty-cycle lengths. The energy cost function was defined as the proportion of the data used when testing the system—using fewer sensors or having longer duty cycles naturally leads to using less data. Since the data is downsampled by half, the baseline data usage is 50%. This function is overly simplistic as the energy consumption details were not given by the dataset authors. Note, however, that the presented methodology could work just as well with any other complex cost function as seen in the Commodity12 example.

### 4.2. Opportunity

Opportunity [50] is a publicly available dataset designed as a benchmark for activity recognition problems. For this dataset the authors recorded four users that followed a loose scenario (waking up, walking around, and preparing breakfast).

Their motion was recorded by 30 sensor clusters—each usually containing an accelerometer, magnetometer, and a gyroscope–placed at different locations on either the participant’s body (on different parts of their arms and legs, in shoes and on the back) or on their surroundings (on the doors, drawers, fridge, on utensils, on glasses, attached to some food, etc.).

Different set of labels were provided to pose different context-recognition problems, out of which we chose the ones where the goal was to recognize the object the user was holding in their right hand. There were 24 possibilities: *bread*, *salami*, *salami2*, *milk*, *cheese*, *sugar*, *glass*, *knife*, *bottle*, *cup*, *spoon*, *plate*, *chair*, *table*, *dishwasher*, *drawer1*, *drawer2*, *drawer3*, *door1*, *door2*, *fridge*, *switch*, *lazychair*, and *none*. The class distribution was highly imbalanced with the *none* being present in 57% of instances while some other contexts, e.g., *lazychair* were present in only a few. Some of the objects were indistinguishable using only the described sensors (opening *door1* is the same as opening *door2* from the point of view of the accelerometer). We joined such classes—both doors into one class, the same for the three drawers, both salamis, both chairs and the fridge with the dishwasher—which resulted in a set of 18 classes.

Our base context-recognition system matched the one from the dataset introductory paper [30]. That is, we used the windows of size 0.5 s, and the features were the mean and the standard deviation for each sensor in these windows. The data was classified using the k-NN classifier and its performance was evaluated using *F-score* as we wanted a balanced accuracy when detecting all classes despite their imbalanced distribution. The (train, validation, test) split was made by taking (50%, 25%, 25%) of data respectively.

As the settings for the optimization of the energy consumption, we used the different sensor subsets and different duty-cycling lengths. Due to the unusually high number of sensors (30 sensor clusters) as well as the high number of contexts (18), we used only some sensor subsets (not all, as usual) as the settings for our methods. These subsets were chosen in the following way: we started with an empty set. Then the sensor cluster that increased the *F-score* on the validation set the most was added to set. This was repeated until adding no single sensor cluster could increase the *F-score*. All resulting sensor subsets—each one cluster bigger than the other—were then used as sensor settings. We then repeated the procedure for each context, this time focusing not on the overall *F-score* but on the ability to detect that particular context only. Again all subsets were added as sensor settings. The idea behind this procedure is that each subset selected is beneficial for recognizing at least one context (and could be potentially used when that context is detected). This eliminated most subsets (159 sensor settings remained) even before starting the optimization process as they would artificially bloat the search space.

Given that the dataset authors did not specify the energy consumption of each sensor, we assumed that it is roughly similar for all of them (this assumption is party validated by the fact that most of them are of the same type). The energy consumption in this case is then directly proportional to the number of sensors active. Consequently, we tried to optimize the average number of sensors active when the context-recognition system is running.

### 4.3. Commodity12

The goal of the Commodity12 project was to create a system that would be used by diabetic patients—it would monitor their activities, helping them to manage their lifestyle more easily. All details can be found in our previous work [51].

As part of this project we monitored 10 volunteers for two weeks using a smartphone and a chest-worn heart monitor. In this time period they could continue their daily lives with no predetermined scenario to follow, but they had to annotate each activity they performed. These activities were then condensed into seven different contexts: *work*, *home*, *transport*, *exercise* (e.g., running and calisthenics), *eating*, *out* (out of house, but not in any of the previous contexts) and *sleep*.

We were then tasked with recognizing these contexts using the sensors they carried—we used the accelerometer, light sensor, barometer, GPS, heart rate, and respiration rate. We also used the list of visible Wi-Fi networks and the location description using Foursquare web service to help with the localization. Bluetooth was not used directly but had to be turned on when we needed the data from the heart-rate monitor. Finally, we used phone’s time as a virtual sensor.

Features were calculated for each minute of the data, and one minute became one learning instance. In this work we recreated the highly personalized setting where the first week of data (from “*user 1*”) was used for training, while the second week was cut in half for testing and validation. This setting was chosen as we hoped it would produce interesting user-specific solutions. Random Forest was determined to be the best performing classification model and was thus chosen to be used in this work on this dataset.

System settings used are again the sensor subsets that is in use and duty-cycling lengths. For this dataset, however, we decided to use a more realistic energy estimation function. We used a multimeter attached to the target device’s battery (Samsung Galaxy S4) and measured the current while having different sensors active. The current is assumed to be proportional to the energy consumption, given constant voltage. When doing the energy estimation, we also had to take into account that energy costs of sensors are not independent. Due to sharing the same hardware, software optimizations, and processor activity, it is often the case that two sensors working in parallel consume less energy than the sum of their individual energy consumption. To take this into account, we manually measured most sensor combinations.

The resulting measurements are presented in full in our previous work [51]. As a quick recap we can mention that using no sensors resulted in the baseline cost of 20 mA, while using all the sensors had a cost of 140 mA. The cheapest “sensor” was time, which provided no additional energy cost, followed by the low frequency accelerometer. The most expensive sensor was unsurprisingly the GPS followed by the Wi-Fi when scanning for visible networks.

### 4.4. E-Gibalec

E-Gibalec was a project aimed at encouraging a more active lifestyle in elementary-school children. The developed mobile application could detect the activities of its user, so they could be rewarded for their active lifestyle. It allowed the child to set its daily movement goals, compete with classmates, and keep upgrading its virtual avatar using the won virtual awards. The whole system is thoroughly described in our two previous works on the topic [52,53].

Ten volunteers (aged 10–12) performed a one-hour-long predetermined scenario, which included different physical activities (*lying*, *sitting*, *slow walking*, *fast walking*, *slow running*, *fast running*, *stretching*, *sit-ups*, *push-ups*, *jumps*, *dodgeball*, *basketball*, *volleyball*, *football*, *cycling*). While a detailed description of their activity was being annotated, the labels were later joined into four elementary activities: *walking*, *running*, *cycling*, *other*.

The smartphone recorded only with the three-axial accelerometer and did so with the sampling frequency of 50 Hz. Data was split into five-second nonoverlapping windows, and features (average of magnitudes, the value of the third quartile, the variance, the coefficient of variation, and the number of peaks) were calculated for each window. To make the train/test/validation split, we used five/two/three volunteers respectively. Random Forest was used as the classifier in this system.

While the reduced computation time and using only one sensor already reduced the energy consumption, we additionally used the presented methodology to optimize two other system settings: the sampling frequency (choosing from: 1, 2, 5, 10, 20, 30, 40, 50) and duty-cycle length.

To determine the energy consumption of the defined settings we started with the empirical measurements that were made as part of the Commodity12 dataset (Section 4.3). From them we determined that the sampling frequency of 5 Hz needs 24 mA of current, while the frequency of 50 Hz needs 46 mA. For the rest of the sampling frequency values we linearly interpolated the energy consumption. This can be justified by the finding of the related work [9,10] that the energy consumption of the accelerometer device increases roughly linearly with its sampling frequency.

When using our energy-reducing methodology on this dataset, a problem arises as the transitions from one activity to another are predetermined and the same for all recorded users. This, however, does not reflect the actual real-life state and may cause the system to overfit to these artificial transitions (e.g., *running* was always followed by *cycling* which always transitioned into *other*). To partly alleviate the issue we first used the expert knowledge to assemble a sensible transition matrix between the activities and then shuffled the dataset with respect to these transition probabilities.

## 5. Results

To get a better intuition for the type of energy-efficient solutions being generated by the methodology we first (Section 5.1) show some sample solutions from each of the methods. Then in Section 5.2 we show the performance improvement achieved when combining two or all three methods. In Section 5.3 we compare our methods to two similar methods from the related work. Finally, in Section 5.4 we discuss the accuracy of the presented mathematical models and their computational speed.

In the presented experiments, the training set is used to train the classification models, the validation set is used for determining setting-to-context assignments and assignments of classifiers in MT trees, and finally the test set is used to estimate performance of all our methods.

### 5.1. Sample Solutions

**Figure 4:**
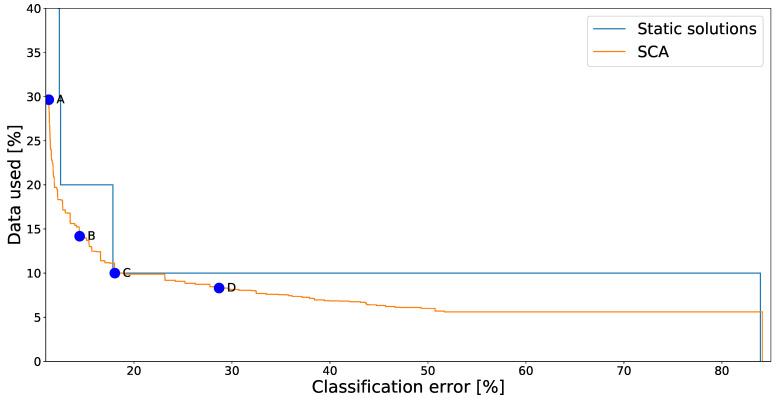
Comparison of assignments, where all contexts have the same sensor combination assigned (Static solutions), to the assignments where active sensors dynamically change based on the last classified context (SCA). Some points of interest are marked with letters A–D. This comparison was done on the SHL dataset.

To showcase solutions from the SCA method we started with an example from the SHL dataset, shown in Figure 4. In this figure we plot the performance of all nondominated solutions found. Solutions displayed in this way are said to form the *Pareto-front approximation* (not the complete Pareto front, because some Pareto-optimal solutions may not have been found).

The settings to be optimized are which sensors to use for each detected context. We compared the solutions found to the “static solutions” where the same subset of sensors was always used. Different points on the graph show different trade-offs between the classification accuracy and energy consumption. In this case the energy consumption was measured based on what percentage of recorded data was used—starting at a baseline of 50%. Since the “ideal” point is the bottom-left one, it is easy to see that dynamic solutions generated by the SCA algorithm consistently outperform the static ones.

Figure 4 also marks some sample solutions. Their details are shown in Table 2.

- Solution A uses the accelerometer regardless of the activity, which is not surprising as the accelerometer is often used in similar context recognition tasks. Additionally it uses the magnetometer whenever a vehicle is detected, which makes it easier for the classifier to distinguish them. Pressure is used when detecting subway and walking (potentially towards it)—as the pressure is different underground. These automatically found sensor assignments thus neatly match the human intuition about the task. Additionally, it is interesting to note that the accuracy of this assignment is 1 percentage point better than of any of the static solutions.
- Solution B uses only the accelerometer during all activities and the magnetometer when a vehicle is detected. While not using three of the five sensors reduced the accuracy, the reduction was quite minimal compared to the solution A (cca. 3 percentage points). Some similar solutions (not explicitly shown here) used the gyroscope instead of the accelerometer when either walking or running—implying that both sensors are roughly equal for the task.
- Solution C uses only the accelerometer for all activities. Its performance therefore matches one of the static solutions.
- Solution D starts abandoning the recognition of some activities altogether by not using any sensors when that activity is detected. The *train* and *subway* activities were the first to be dropped, probably due to having the worst recognition accuracy even when appropriate sensors were used. Note that if no sensors are active, the majority classifier is used—classifying everything as *car*.

As an additional example of SCA solutions, we show two sample solutions from the Commodity12 dataset (Table 3). Solution 1 uses GPS whenever the user is detected as potentially “outdoors” (*Out*, *Eating*, *Exercise*, *Transport*), sound to differentiate if the user is sleeping or “active” and accelerometer when *Exercise* (and somewhat curiously *Eating* is detected). Solution 2 is much more streamlined and more energy-efficient: relying on the current time and the visible Wi-Fi spots to differentiate between most contexts.

**Figure 5:**
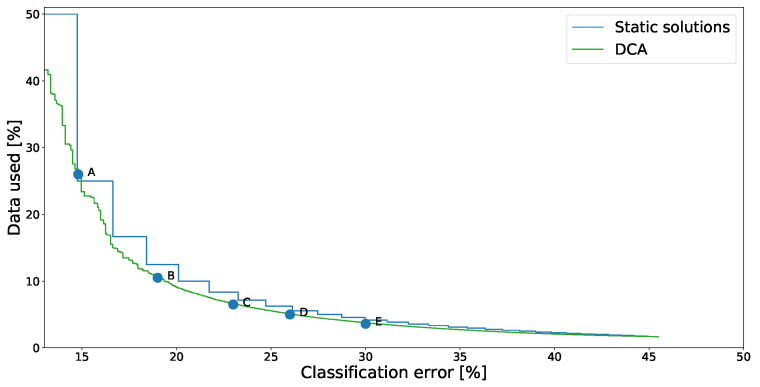
Comparison of assignments on the SHL dataset where all contexts have the same duty-cycle length assigned (Static solutions) to the assignments where duty-cycles lengths dynamically change based on the last classified context (DCA).

Next, we show DCA solutions for the SHL dataset (Figure 5) compared to “static solutions”, where all contexts use the same duty-cycle length. Again, some sample solutions are marked with letters (A–E) and their exact parameters are listed Table 4. They show that activities *run*, *walking*, and *still* have short duty cycles assigned. Intuitively, *run* is a relatively short activity, while *walking* and *still* activities act as hubs from which other activities often transition. The *car* and *train* activities are the longest on average and have correspondingly long duty cycles. Interestingly, *bus* has longer duty cycles assigned than *train*, maybe due to the fact that *bus* classifications are more likely to be correct than *train* classification.

**Figure 6:**
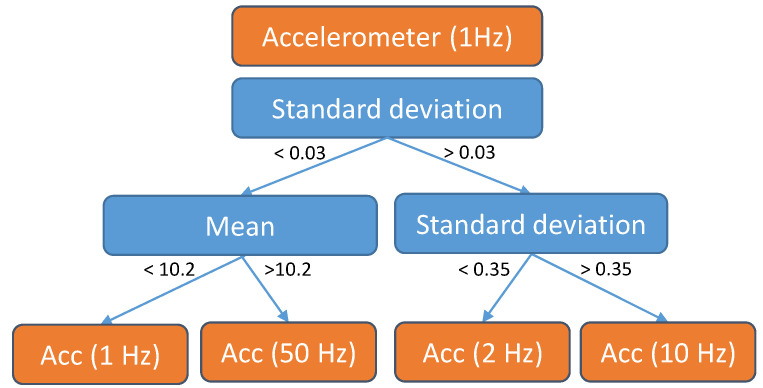
An example of a MT from the E-Gibalec dataset. Orange nodes prescribe the sensor settings required, while the blue nodes represent features used to determine these settings.

Finally, we show an example of a MT from the E-Gibalec dataset (Figure 6). This tree always uses the accelerometer sensor with the sampling frequency of 1 Hz but can prescribe a higher sampling frequency depending on the feature values.

The left most leaf node is reached in cases where no motion is being detected, and the lowest sampling frequency is thus chosen. The following leaf node (going from left to right) represents the case where the user is either walking or cycling—the two hard to distinguish contexts—and the highest possible sampling frequency is chosen. The last two nodes represent values that happen when walking and running respectively. Running needs higher sampling frequency as it is the fastest moving motion recorded.

Another example of a MT (although simplified) from the Commodity12 dataset was shown in Figure 2 in Section 3.3.

### 5.2. Method Performance and Comparison

**Figure 7:**
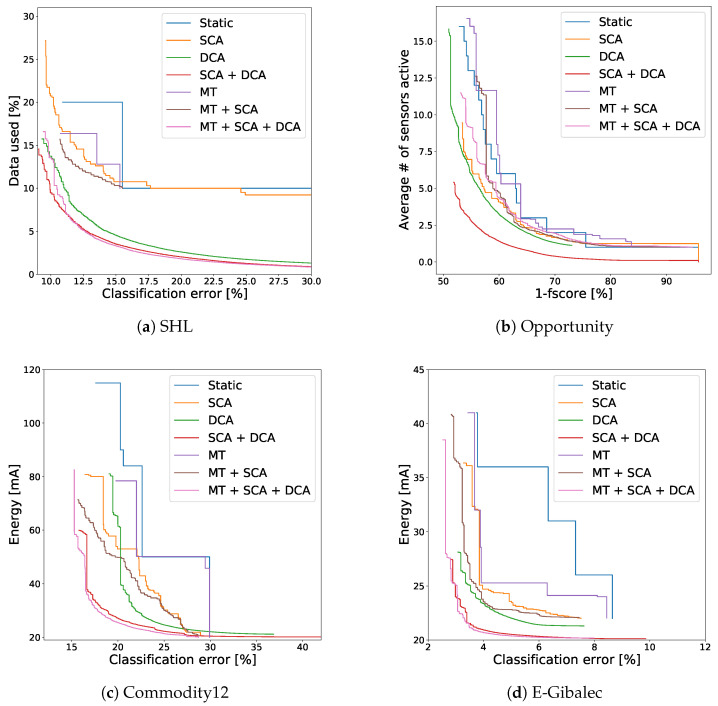
Comparison of all our methods and their combinations. All tests were performed on four datasets.

The performance of all the presented methods and their combinations is shown in Figure 7 for each dataset. In all cases the static solutions (always use the same setting for each context) are the worst performing one. The DCA method usually outperforms the SCA method, showing the importance of the duty-cycle settings in many context-recognition tasks. Importantly, after the MT method is combined with the SCA method, their joint performance improves. The same goes for the SCA + DCA combination.

The benefit of combining all three methods, however, is less clear. In the case of E-Gibalec (Figure 7d) and Commodity12 (Figure 7c), the three-method combination dominates all others. In the case of SHL (Figure 7a), it dominates only on the “right”—lower accuracy, greater energy efficiency—part of the solution space, compared to the SCA + DCA combination. Finally, on the Opportunity dataset (Figure 7b), the performance substantially decreases when the three-method combination is used. This could be by chance, but a reasonable explanation could lie in the number of contexts. The E-Gibalec dataset has only four different contexts, thus SCA can only work with coarse granularity and is improved by the MT method. The Opportunity dataset, on the other hand, contains 18 contexts and by splitting them based on feature values might create too large an assignment space, given the dataset size—leading to overfitting. An empirical argument for the overfitting is that the MT solutions on the Opportunity dataset are actually worse than the static solutions—which in theory should not happen as placing the same classifier into all leaf nodes effectively results in a solution that is equivalent to the static one. Thus we should expect at least equal if not greater performance from the MT solutions. Due to overfitting, however, the increased performance only showed on the training set but not on the testing set.

Another explanation comes from the delayed sensor switching introduced by combining the MT and DCA methods (Section 3.4.2). After testing the same solutions, but with the delay artificially removed (not feasible in practice), the results improved—and started to dominate all the solutions in the SHL dataset (but were still worse on the Opportunity dataset). We believe that the two listed causes result in the inconsistent performance of the three method combination but will need additional testing in other domains to confirm it.

### 5.3. Comparison to Related Work

Finally, we compare our methodology against two methods from the related work. These two methods were chosen as they optimize similar sensor settings as our methods and are described in enough detail so we could fully reimplement them. In addition, the first one is comparable to the SCA method (in terms of generality, sensor settings used, etc.) and the second one to the DCA method.

The first method from the related work (*Activity recognition for creatures of habit*) [29] decides on which sensor subset to use based on which context (or which subset of contexts) is most likely to appear next. The likelihood is estimated by assuming the context sequence is Markov (the same assumption is made in our approach). The subset used is the one that is good for recognizing both the current and likely next contexts. For this approach to work, the system designer must choose two parameters: the acceptable accuracy loss [0–100%] and the number of likely contexts predicted [1–$\left|C\right|$]. Authors do not specify how to determine the parameters, so we conducted a thorough grid search of values in the previously specified range. Grid search also enables us to select nondominated solutions and construct the resulting Pareto front approximation.

The second method (*Episodic sampling*) [32] is a method that changes the duty-cycle length based on the frequency of context transitions. If the context remains unchanged, the length is increased by *a*, if not, the length is divided by *b*. The *a* and *b* are the parameters of the system and were determined by a grid search of sensible values (a∈ [0–15], b∈ [0–1]). Again all nondominated solutions were kept.

Neither of the proposed methods provide a model for the solution quality estimation and thus rely on the simulation for making such estimation.

**Figure 8:**
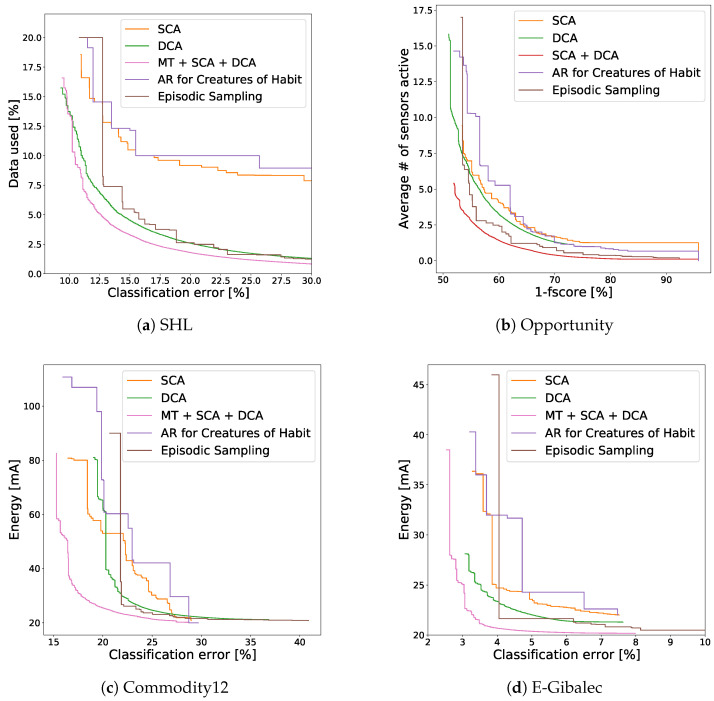
Comparison of our methods to two alternatives from the related work. All tests were performed on four datasets.

The results are shown in Figure 8. The fairest comparison is the SCA method against the “*Activity recognition for creatures of habit*” and the DCA method against the “*Episodic sampling*”. In most cases solutions from our methods dominate those from related work. The exception is again the Opportunity dataset (Figure 8b), where *Episodic sampling* overperforms. In all cases our method combination achieves better results than any of the tested methods from the related work. The number of nondominated solutions and their hypervolume—the area that is being dominated by the solutions—for all methods is listed in Table 5.

### 5.4. Method Time and Accuracy

The key component of the proposed methodology is using two mathematical models (one for the SCA method and the other for the DCA method) that can predict the performance of any assignment. This avoids the need for testing assignments empirically, by going through instances one-by-one, classifying them and then simulating different settings based on the current classification.

First, we show the difference in speed between the model and the previously described simulation like approach (Table 6). The times reported are the average over a 100 random assignments. In all cases the SCA and DCA models require similar time to compute, that is in most cases roughly 100-times faster than the simulation. The only exception is the SCA model on the Opportunity dataset, where the model is only two times faster. The slow performance can be explained by the large number of contexts in this dataset, as the most computationally expensive operation is the multiplication of c∗c matrices, where *c* is the number of contexts.

The overall time for all steps required (running the optimization for the SCA + DCA method, generating MTs, combining the methods etc.) varies significantly based on the number of contexts and settings—in our example datasets it ranged from minutes to an hour on standard hardware. It is of note, however, that all the computation is front-loaded. Once the solutions are created and selected, the real-time requirements on the context-recognition device are negligible and can be implemented with a few “if” statements.

Next, we had to validate that our predictions of assignment performance are indeed accurate. In this experiment we evaluated 100 random assignments and used the simulation performance as the ground truth. Note, this model accuracy should not be confused with the classifier accuracy of the context recognition system. For example, if our model predicts that the context recognition system performs at 81% accuracy, but the simulation shows that actual accuracy is 83% then our model made a 2 percentage point (p. p.) error. This is similar when predicting energy consumption. The results are shown in Table 7.

The error made varies from dataset to dataset but it is roughly 1 percentage point on average, making it accurate enough for our use. For comparison, we also created a simple model described in Section 3.1.1 that is in a spirit similar to what some methods from related work [15,28] implicitly use. Its error is significantly higher than the one from our methods.

## 6. Conclusions

In this work we presented three different methods that can increase the energy-efficiency of a context recognition system. In addition, we showed how to combine them to improve energy-efficiency even further.

The core of the methodology consists of mathematical models that can predict the performance of a system that is adapting its settings based on the last recognized context. These models were shown to be accurate—with an error of 1–2 percentage points on average—and roughly a hundred times faster than simulation in most cases. This allowed us to use multiobjective optimization to find good solutions.

The biggest contribution in comparison with our previous work is the addition of the MT component and the combining of our methods. The MTs were shown to increase the energy efficiency in almost all cases—their contributions seemingly being linked to the number of contexts in the domain (the lower the number of contexts, the bigger the contribution). Combining the SCA and DCA method, on the other hand, yielded improvements to the solutions in all cases.

The approach was tested on four different, complex datasets and achieved consistently good results. All of the presented methods return the whole Pareto-front approximation of different trade-offs between the classification quality and energy consumption. As an example of some solutions found: for the SHL dataset we were able to use only 5% of the available data (by using lower frequency, duty cycling, and using a sensor subset) and in exchange sacrifice less than four percentage points of the accuracy. For the Opportunity dataset we were able to find a solution that uses five sensors on average (instead of the original 30) and in exchange loses one percentage point of F-score. For the Commodity12 dataset, we decreased the smartphone’s energy consumption from 123 mA to 40 mA. In exchange we lost less than 1 percentage point of accuracy. Finally, for the E-Gibalec dataset, the energy was reduced from 46 mA to 24 mA also for less than one percentage point of accuracy lost.

The methods outperformed two state-of-the-art methods from the related work—both when compared to our methods used individually but especially when our methods were combined.

The presented methods are very general and should be applicable to most context recognition systems. They require no expert knowledge (with the exception of selecting possible settings) and almost no hand picked parameters. In the E-Gibalec dataset example, the system designer only had to determine that the possible settings are the different frequencies in 1–50 Hz range (with a provided energy estimate for them) and duty-cycle lengths with the upper sleep limit of 30 classification windows of 5 s—all other steps were automated. The method implementation is openly available in the form of a Python library [8] and we hope it could be of use to designers of context-recognition systems.

The most notable limitation of our methodology is its inability to tackle regression problems (e.g., the location coordinates of the user). The is due to the SCA and DCA methods using a discrete context, i.e., the classification class, to adapt the sensor settings. In order to use these methods on a regression problem, either the class or feature values would have to discretized and then used as the “context”—a potential research problem left for the future work. On the other hand, the MT method could easily be adapted by using regressors in the decision-tree leaves instead.

As part of our future work we intend to use this framework in our upcoming context-recognition projects and implement it on real devices—and thus further validating its practical usefulness.

## Figures and Tables

**Table 1:** The number of classes, possible system settings (duty cycling not included) for each of the datasets, and the magnitude of possible setting-to-context assignments. The type of system settings being optimized is also listed.

	# Classes	Setting Type	# Settings	# Assignments
SHL	8	Sensors used, duty cycling	32	≈$10^{13}$
Opportunity	18	Sensors used, duty cycling	159	≈$10^{40}$
Commodity12	7	Sensors used, duty cycling	1024	≈$10^{21}$
E-Gibalec	4	Sampling frequency, duty cycling	8	≈$10^{4}$

**Table 2:** The sensors used when the corresponding activity is detected for each of the four solutions marked in Figure 4. Sensors are abbreviated: acc—accelerometer, mag—magnetometer, ori—orientation sensor, gyr—gyroscope, bar—barometer. Both accuracy and energy costs (measured in the percentage of data used) are provided as well.

	Solution A	Solution B	Solution C	Solution D
Accuracy [%]	88.7	85.5	82.0	71.3
Energy [%]	29.6	14.2	10	8.3
Bike	acc, gyr, ori	acc	acc	acc
Bus	acc, mag, gyr	acc, mag	acc	acc
Car	acc, mag	acc	acc	acc
Run	acc, gyr	acc	acc	gyr
Still	acc, gyr, ori	acc	acc	acc
Subway	acc, mag, bar, ori	acc, mag	acc	\
Train	acc, mag, gyr, ori	acc, mag	acc	\
Walking	acc, bar	acc	acc	acc

**Table 3:** The sensors used when the corresponding context is detected for two sample solutions. Some sensors are abbreviated: sqr—FourSquare webservice, wifi—Wi-Fi, acc—(accelerometer, pressure, light), hr—(heart rate, respiration rate). Multiple sensors are listed in brackets as they all share a cost and are thus either all included or excluded.

Points A	Solution 1	Solution 2
Accuracy [%]	85.2	80.7
Energy [mA]	65.6	38.6
Eating	acc, time, gps	time, gps
Exercise	acc, time, gps, wifi	time, wifi
Home	sound, time, gps	time, wifi
Out	hr, gps, wifi	acc, time, gps
Sleep	sound, time	sound, time
Transport	time, gps	time, wifi
Work	wifi	wifi

**Table 4:** Sample solutions returned by the DCA method for the SHL dataset. For each activity the assigned duty-cycle length is given (the length of the sleeping phase of the cycle). In addition, both the accuracy and energy costs are listed. Duty-cycle length of 0 implies that duty cycling should not be used.

	Bike	Bus	Car	Run	Still	Subway	Train	Walk	Acc. [%]	Data Used [%]
A	0	2	5	2	1	1	0	1	85	26
B	4	4	7	4	3	5	4	3	81	10
C	4	10	13	3	5	7	7	5	77	7
D	7	13	13	6	6	10	11	6	74	5
E	11	13	20	7	8	14	15	9	70	4

**Table 5:** Hypervolume calculated on the nondominated sets as returned by all our methods and the two methods from the related work. Both objectives were normalized to the [0–1] interval based on their max and min values and the reference point was set to (1.1, 1.1), making the ideal hypervolume 1.21. The best method based on the hypervolume is bolded for each dataset. In addition, we list the size of each nondominated set. ES—Episodic sampling, CoH—Activity recognition for creature of habit.

Method	SHL	Opportunity	Commodity12	# E-Gibalec
	**Hypervolume**			
Static	0.957	0.579	0.956	1.034
SCA	1.974	0.590	0.979	1.118
DCA	1.074	0.608	0.992	1.084
SCA + DCA	**1.097**	**0.628**	1.026	1.169
MT	0.853	0.560	0.957	1.044
MT + SCA	0.993	0.562	1.010	1.090
MT + SCA + DCA	1.085	0.584	**1.033**	**1.172**
CoH	0.993	0.596	0.980	0.983
ES	1.067	0.607	0.967	1.097
	**# Solutions**			
Static	4	17	5	7
SCA	92	212	251	84
DCA	712	996	1260	289
SCA + DCA	1019	2096	2050	410
MT	3	71	29	8
MT + SCA	71	445	251	76
MT + SCA + DCA	920	778	562	313
CoH	25	36	13	14
ES	34	44	22	21

**Table 6:** Time of evaluation [ms] of the mathematical models compared the time of the simulation. Test was repeated and averaged over 100 random assignments.

	Model	Simulation
SHL–SCA	7	440
SHL–DCA	1.3	420
Opportunity–SCA	158	263
Opportunity – DCA	18	628
Commodity–SCA	5	121
Commodity–DCA	0.7	225
E-Gibalec–SCA	0.4	90
E-Gibalec–DCA	0.3	140

**Table 7:** Difference (mean absolute error) between the predicted accuracy/energy and the one calculated in a simulation for the SHL dataset. The experiment was repeated for both Simple, SCA, and DCA method, for 100 random assignments. The difference in accuracy (o F-Score in the case of Opportunity) was measured in percentage points, while the difference in energy was measured in the percentage of maximal energy used in the respective dataset.

	Accuracy	Energy
SHL–SCA	0.8	0.1
SHL–Simple	13.4	1.5
SHL–DCA	2.8	0.5
Opportunity –SCA	1.0	0.08
Opportunity –Simple	9.1	0.6
Opportunity –DCA	3.2	0.5
Commodity–SCA	2.4	1.3
Commodity–Simple	5.2	3.2
Commodity–DCA	0.7	0.2
E-Gibalec–SCA	0.6	0.08
E-Gibalec–Simple	2.9	0.3
E-Gibalec–DCA	0.04	0.12

**Figure A1:**
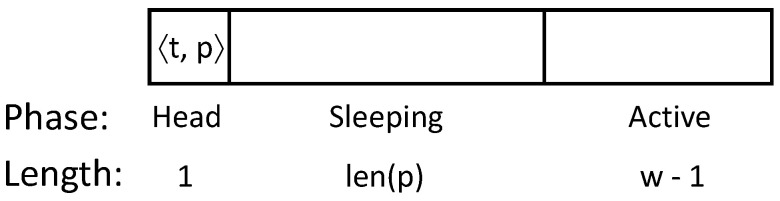
Structure of a 〈t,p〉 duty cycle. It starts with the context *t* classified as *p*, followed by the sleeping phase (sensors not working) where all contexts are classified as *p*, and an active phase (sensors working). The last period in the active phase is the head of the next cycle.

## Data Availability

Publicly available datasets were analyzed in this study. This data can be found here: http://www.shl-dataset.org/activity-recognition-challenge and https://archive.ics.uci.edu/ml/datasets/opportunity+activity+recognition/.

## Abbreviations

The following abbreviations are used in this manuscript:

SCA	Setting-to-Context Assignment
DCA	Duty-cycle-to-Context Assignment
MT	Model Trees

## Appendix A. Using Markov Chains in the SCA Method

We start by evaluating the performance of the system using each of the settings (each setting is tested independently, with no switching between them). This is done by simulating a setting, e.g., using data from only a few sensors or downsampling data to another frequency, etc. and then determining the confusion matrix of such a system. In addition, we need the transition probabilities $T(c_{i},c_{j})$ from each context to each other, which can easily be inferred from the dataset.

Having both the confusion matrices when a single setting is used as well as transition probabilities, we can estimate the performance of the system when settings are dynamically changing based on the recognized context. This is done by first appropriately defining Markov states (Section 3.1.2) and then calculating the transition probabilities between these Markov states. This is done by using Equation (A1)—which is simply stating that to get to the state $\langle c,c^{\prime }\rangle$ the context must really change to *c* and the system must classify it into $c^{\prime }$.

(A1) $$P\left(\langle c_{1},c_{2}\rangle \to \langle c_{3},c_{4}\rangle \right)=T\left(c_{1},c_{3}\right)CM_{a(c_{2})}\left(c_{4}|c_{3}\right)$$

$P(\langle c_{1},c_{2}\rangle \to \langle c_{3},c_{4}\rangle )$—the probability of a transition from state $\langle c_{1},c_{2}\rangle$ to state $\langle c_{3},c_{4}\rangle .$

$T(c_{1},c_{3})$—the probability in the dataset that the next context will be $c_{1}$ given that the current one is $c_{3}$.

a(c)—the setting assigned to context *c*.

$CM_{s}\left(c_{4}|c_{3}\right)$—the probability that the classifier that works with setting *s* will classify an instance to $c_{4}$, if the true context is $c_{3}$.

To calculate the “steady-state” of the Markov chain we have to solve the equation xP=x, where *P* is the matrix of transition probabilities between Markov states and *x* is the desired vector, where each component represents the proportion of time spent in each of the states (given that the system runs for long enough time).

## Appendix B. Calculating the Performance of a DCA Assignment

In this appendix we summarize the steps required to mathematically asses the performance of a context recognition system that uses duty cycling.

### Appendix B.1. Duty-Cycle Types

We start by splitting duty cycles into different types. The duty cycle type depends both on the context that appears in the last active period (marked as “head” in the Figure A1 as well as on the context that period was classified into. Indices of these contexts are subsequently named *t* and *p*, respectively. If the last active period before sleeping is *walk* that is classified as *run* we have a 〈walk,run〉 cycle. The first component of the cycle allows us to calculate the likely context transitions, while the second one determines the length of the sleeping phase.

For each of those cycle types, we can use the following equations to estimate how much of each context appears in both the sleeping (A4) and active (A5) phase. We start by the state distribution in the *head* of the 〈t,p〉 cycle–by definition the state there is known and thus the state distribution is $e_{t}$. For each next time period we can estimate the state distribution by multiplying the previous distribution with the transition matrix *T*. Having the context probability at each time step, their total expected number is simply the sum of their probabilities at each step.

(A2) $$e_{i}={\left[0,\ldots ,0,\underset{i}{\underset{︸}{1}}0,\ldots ,0\right]}^{T}$$

(A3) $$P_{t,i}=e_{t}T^{i}$$

(A4) $$ES_{t,p}=\sum_{i=0}^{len(p)}P_{t,i}$$

(A5) $$EA_{t,p}=\sum_{i=len(p)+1}^{len(p)+a-1}P_{t,i}$$

$e_{i}$—the *i*-th base vector. It represents the context distribution in the first period of a cycle.
*T*—the transition matrix, $T_{i,j}$ represents the probability of the next context $c_{j}$, given the current context $c_{i}$. Easily obtained from the dataset.
$P_{t,i}$—the probability distribution of contexts after *i* steps, given the current context $c_{t}$. Vector of size |C|.
$ES_{t,p}$—the average number of contexts that appear while sleeping, given that this sleeping phase started with the true context $c_{t}$ that was classified as $c_{p}$. Vector of size |C|.
$EA_{t,p}$—the average number of contexts that appear while active, given that this cycle started with the true context $c_{t}$ that was classified as $c_{p}$. Vector of size |C|.

### Appendix B.2. Distribution of Duty-Cycle Types

In the previous section we showed how a specific 〈t,p〉 cycle behaves, given we know its type. The next point of interest is determining the expected proportion of each cycle type. To do so we first calculate the probability that a cycle of type $\langle t_{1},p_{1}\rangle$ follows a cycle of type $\langle t_{2},p_{2}\rangle$ for each *t* and *p*. Intuitively, this happens if $t_{1}$ transitions to $t_{2}$ after $len(p_{1})+w$ steps (length of the cycle) and if the $t_{2}$ is classified as $c_{2}$. The probability of that happening is expressed in Equation (A6).

(A6) $$P\left(\langle t_{1},p_{1}\rangle \to \langle t_{2},p_{2}\rangle \right)={\left(P_{t_{1},len\left(p_{1}\right)+a}\right)}_{t_{2}}CM_{t_{2},p_{2}}$$

${\left(P_{i,j}\right)}_{k}$—the *k*-th component of the vector $P_{i,j}$.

$P(\langle t_{1},p_{1}\rangle \to \langle t_{2},p_{2}\rangle )$—the probability of the next cycle type being $\langle t_{2},p_{2}\rangle$, given the current cycle type $\langle t_{1},p_{1}\rangle$. This happens if the last context of the previous cycle is $t_{2}$, classified as $p_{2}$.

Having the transition probabilities, we can use the basic Markov-chain calculus to calculate the steady state in the similar way as in Section 3.1.2. The steady state corresponds to the desired cycle-frequency proportions *D*.

### Appendix B.3. System Evaluation

Combining the results from the previous two sections, we can estimate the performance of the system in the form of its confusion matrix (A7) and its energy gain (A8). Energy gain is defined as the average number of sleeping periods for each active one. If the sensors are on half the time, Gain=1.

The intuition behind calculating the energy gain is simple. We know how long the cycle is given its type. We also know the distribution of each type. The double loop in Equation (A8) simply iterates over all cycle types and sums the two terms together. The calculation of the new confusion matrix is slightly more complex and can be split into two parts: performance while sleeping and the performance during the active phase. While sleeping in a cycle of type 〈t,p〉, all the contexts are classified as *p* while the proportion of the contexts actually appearing in that period is given by the ES. On the other hand, while active we know the proportion of appearing contexts (EA) as well as the misclassification of each context. This is formalized in Equation (A7).

(A7) $$CM_{t,p}^{*}=\sum_{i=1}^{\left|C\right|}D_{i,p}{\left(ES_{i,p}\right)}_{t}+\sum_{i=1}^{\left|C\right|}\sum_{j=1}^{\left|C\right|}D_{i,j}{\left(EA_{i,j}\right)}_{t}CM_{t,p}$$

(A8) $$Gain=\frac{1}{w}\sum_{i=1}^{\left|C\right|}\sum_{j=1}^{\left|C\right|}len\left(j\right)D_{i,j}$$

*D*–the distribution of the cycle types. $D_{i,j}$ is the proportion of 〈i,j〉 cycles.
$CM_{t,p}^{*}$–element (t,p) of the confusion matrix of the system that employs duty cycling with given parameters. The first part of the equation is the performance in the sleeping phase, the second in the active phase. Note: The sum of all the elements in this new matrix is different than in the original CM matrix; this can be easily solved with normalization if need be.
Gain—the proportion of sleeping periods compared to the active ones.
